# Advanced Enzymology, Expression Profile and Immune Response of *Clonorchis sinensis* Hexokinase Show Its Application Potential for Prevention and Control of Clonorchiasis

**DOI:** 10.1371/journal.pntd.0003641

**Published:** 2015-03-23

**Authors:** Tingjin Chen, Jinyun Yu, Zeli Tang, Zhizhi Xie, Zhipeng Lin, Hengchang Sun, Shuo Wan, Xuerong Li, Yan Huang, Xinbing Yu, Jin Xu

**Affiliations:** 1 Department of Parasitology, Zhongshan School of Medicine, Sun Yat-sen University, Guangzhou, Guangdong, China; 2 Key Laboratory for Tropical Disease Control, Sun Yat-sen University, Ministry of Education, Guangzhou, Guangdong, China; University of Melbourne, AUSTRALIA

## Abstract

**Background:**

Approximately 35 million people are infected with *Clonorchis sinensis* (*C*. *sinensis*) globally, of whom 15 million are in China. Glycolytic enzymes are recognized as crucial molecules for trematode survival and have been targeted for vaccine and drug development. Hexokinase of *C*. *sinensis* (*Cs*HK), as the first key regulatory enzyme of the glycolytic pathway, was investigated in the current study.

**Principal Findings:**

There were differences in spatial structure and affinities for hexoses and phosphate donors between *Cs*HK and HKs from humans or rats, the definitive hosts of *C*. *sinensis*. Effectors (AMP, PEP, and citrate) and a small molecular inhibitor regulated the enzymatic activity of r*Cs*HK, and various allosteric systems were detected. *Cs*HK was distributed in the worm extensively as well as in liver tissue and serum from *C*. *sinensis* infected rats. Furthermore, high-level specific IgG1 and IgG2a were induced in rats by immunization with r*Cs*HK. The enzymatic activity of *Cs*HK was suppressed by the antibody in vitro. Additionally, the survival of *C*. *sinensis* was inhibited by the antibody in vivo and in vitro.

**Conclusions/Significance:**

Due to differences in putative spatial structure and enzymology between *Cs*HK and HK from the host, its extensive distribution in adult worms, and its expression profile as a component of excretory/secretory products, together with its good immunogenicity and immunoreactivity, as a key glycolytic enzyme, *Cs*HK shows potential as a vaccine and as a promising drug target for Clonorchiasis.

## Author Summary

Clonorchiasis, caused by *Clonorchis sinensis* (*C*. *sinensis*) infection, is a kind of neglected tropical disease. There are still few effective measures to prevent clonorchiasis. As in other helminthes, hexokinase (HK) has been well characterized as a target for vaccine and drug development. In the current study, we identified differences in spatial structure between *Cs*HK and HKs from the definitive *C*. *sinensis* hosts, humans and rats. We also characterized the substrate specificity and allosteric regulation of r*Cs*HK in detail. The distribution of *Cs*HK in the worm and in the liver tissue and serum from *C*. *sinensis* infected rats were confirmed. Furthermore, a high-level specific antibody in rat was induced by immunization with r*Cs*HK. The enzymatic activity of *Cs*HK was suppressed by the antibody in vitro. Additionally, the survival of *C*. *sinensis* was inhibited by the antibody in vivo and in vitro. Our study shows that *Cs*HK has vaccine potential and is a promising drug target for Clonorchiasis.

## Introduction

Clonorchiasis, induced by *Clonorchis sinensis* (*C*. *sinensis*) infection, is a major public health problem in Southeast Asian countries including China, Korea, Taiwan, and Vietnam. Approximately 35 million people are infected with this neglected fluke globally, of whom 15 millions are in China [1]. The World Health Organization (WHO) announced in 2009 that *C*. *sinensis* infection is one of the biological agents that can induce cholangiocarcinoma [2]. In spite of its public health threat, there are still few effective measures to prevent this neglected tropical disease. Humans can be infected with *C*. *sinensis* by ingestion of raw or undercooked freshwater fish with metacercariae. The metacercariae of *C*. *sinensis* excyst in the duodenum, then migrate into hepatic bile ducts where the flukes mature into adult worms [3]. During the long term of parasitism, the worms continuously release excretory/secretory products (ESPs), a cocktail of hundreds to thousands of bioactive proteins. As molecules involved in the interaction between the parasite and host, ESPs have been well characterized to be targets for vaccine and drug development [4–7].

Glycolytic enzymes such as enolase [4, 8] and phosphoglycerate kinase [9, 10] are recognized as crucial molecules for trematode survival, and they have been targeted for vaccine and drug development. Hexokinase (HK) (ATP: D-hexose-6-phosphotransferase, EC 2.7.1.1) is the first key regulatory enzyme of the glycolytic pathway [11]. In other helminthes such as *Brugia malayi* (*B*. *malayi*) [12], *Haemonchus contortus* [13], and *Schistosoma mansoni* (*S*. *mansoni*) [14–16] HKs have been well characterized as potential targets for vaccine and drug development. In our previous study, the sequence, structure, and enzymatic properties of HK from *C*. *sinensis* (*Cs*HK) were confirmed, and its molecular characteristics including molecular mass, mRNA and protein levels during different life stages of *C*. *sinensis* were determined [17]. These studies are cornerstones for our current study.

In the present study, we compared the putative spatial structure of *Cs*HK with HKs from definitive hosts of *C*. *sinensis*. The effects of a small molecule inhibitor on the enzyme kinetics of recombinant *Cs*HK (r*Cs*HK) and the immunological characteristics and immune protective efficacy of r*Cs*HK were investigated in detail. Our results indicate that *Cs*HK may be a promising candidate for development of vaccines and drugs against *C*. *sinensis* infection.

## Methods

### Ethics statement

All animals used in the present study were purchased from the animal center of Sun Yat-sen University and raised carefully in accordance with National Institutes of Health animal care and ethical guidelines. All experimental procedures were approved by the Animal Care and Use Committee of Sun Yat-sen University (Permit Numbers: SCXK (Guangdong) 2009–0011). The ethical approval for human sera was granted from the Centers for Disease Control and Prevention of Guangxi Zhuang Autonomous Region, China. All human serum samples used in this study were anonymized.

### Preparation of parasites, ESPs of *C*. *sinensis* (*Cs*ESPs) and antiserum against *Cs*ESPs/r*Cs*HK

Metacercariae of *C*. *sinensis* were isolated from experimentally infected freshwater *Ctenopharyngodon idellus* fish in our laboratory pool [18]. Each Sprague-Dawley (SD) rat was orally infected with 50 metacercariae. At 8 weeks after infection, the rats were sacrificed and *C*. *sinensis* adults were recovered from the livers.

*Cs*ESPs and rat anti-*Cs*ESPs serum were obtained as described before [4]. Purified r*Cs*HK was obtained in our previous study [17]. Purified r*Cs*HK (200 μg) emulsified with an equal volume of complete Freund’s adjuvant (Sigma, USA) was injected subcutaneously into SD rats. Two boosters of 100 μg r*Cs*HK with an equal volume of incomplete Freund’s adjuvant (Sigma, USA) were given at 2-week intervals. The pre-immune sera were collected prior to the first injection. The immune sera were collected at 2-week intervals from 0 to 12 weeks.

### Comparison of putative spatial structure of *Cs*HK with HKs from definitive hosts of *C*. *sinensis*, human and rat

As the amino acid sequence of *Cs*HK shares 69% identical residues with the *S*. *mansoni* sequence [17], the putative tertiary structure of *Cs*HK was constructed based on that of HK from *S*. *mansoni* (*Sm*HK, Protein Data Bank, PDB: 1BDG_A) using SWISS-MODEL and viewed by Swiss-Pdb Viewer [17, 19]. Structural models of *Cs*HK were superposed with closed-form human glucokinase (hHK-IV, PDB: 1V4S_A) [20] and the N-terminal half of closed-form rat hexokinase-1 (rHK-In, PDB: 1BG3_B) [21]. The allosteric sites in closed-form hHK-IV [20] and *Cs*HK were compared. The glucose 6-phosphate (G6P) binding sites in *Cs*HK were compared to that of rHK-In [21]. The accession numbers/ID numbers for genes and proteins mentioned in the text are listed in S1 Table.

### Effects of phosphate donors, effectors and a small molecule inhibitor on the enzyme kinetics of r*Cs*HK

The enzymatic activity of HK was assayed as described using a coupled reaction [17, 22]. A 200-μL aliquot of reaction mixture included 3 mM glucose, 3 mM ATP, 15 mM MgCl_2_, 0.5 mM nicotinamide adenine dinucleotide phosphate (NADP), 0.3 U of yeast glucose 6-phosphate dehydrogenase (G6PD) Type VII, and 100 mM Tris-HCl (pH 8.5). Reduced NADP (NADPH) formation by G6P dehydrogenation was monitored at 340 nm in a microplate reader (SpectraMax M5, Molecular Devices, USA). All enzymatic reagents were purchased from Sigma-Aldrich (USA).

To determine the kinetic parameters of r*Cs*HK, the substrate (ATP, CTP, GTP, ITP, TTP, UTP, or glucose) concentrations were varied from 0.05 to 3 mM. Effectors such as AMP (0–5 mM), phosphoenolpyruvate (PEP, 0–10 mM), and citrate (0–10 mM) were added to the reaction mixture to investigate their effects on enzymatic activity of r*Cs*HK, as was 2-phenyl-1, 2-benzisoselenazol-3(2H)-one (EbSe, a small molecular inhibitor, 0–100 μM). Note that EbSe was found to be ineffective in a counterscreen for inhibition of G6PD [23].

### Western blotting analysis

Purified r*Cs*HK protein (2 μg) or *Cs*ESPs (30 μg) was subjected to 12% SDS-PAGE and then electrotransferred onto a polyvinylidene difluoride (PVDF) membrane (Whatman, UK) at 100 V for 60 min in a Trans-Blot transfer cell (Bio-Rad, USA). The PVDF membranes were blocked with 5% (w/v) skimmed milk in phosphate buffer saline (PBS, pH 7.4) overnight at 4°C and then probed with serum from *C*. *sinensis* infected humans/rats, healthy people, r*Cs*HK immunized rats or pre-immune rats for 2 h at room temperature (RT). All the sera were at the same dilution of 1:200. After washing with PBS three times, the membranes were then incubated in horseradish peroxidase (HRP)-conjugated goat anti-human/rat IgG (1:2,000 dilution, Protein tech., USA) for 1 h at RT. Both the primary and secondary antibodies were diluted with 0.1% BSA in PBS (pH 7.4). After washing five times, the membranes were developed with diaminobenzidine (DAB, Boster, China) reagent according to the manufacturer’s instructions.

### Immunolocalization of *Cs*HK in *C*. *sinensis* and in liver tissue from infected rats

Adult worms and metacercariae of *C*. *sinensis* and liver tissue from infected rats were fixed with formalin, embedded with paraffin wax and sliced into 4 μm-thick sections. The sections of adult worms and metacercariae were deparaffinized in xylene, hydrated in gradient alcohol and then blocked with normal goat serum for 2 h at RT. The sections were incubated in mouse anti-r*Cs*HK serum (1:100 dilution) previously obtained [17] in a humid chamber at 4°C overnight. Serum from a pre-immune mouse was employed as a negative control. After successively washing three times with PBS containing 0.05% Tween-20 (PBST, pH 7.4) and two times with PBS, the sections were incubated with Cy3-conjugated goat anti-mouse IgG (1:400 dilution, Molecular Probe, USA) for 1 h at RT in the dark. BSA (0.1%) in PBS was employed as dilution buffer. The sections were subsequently imaged under a fluorescence microscope (Leica, DMI3000B, Germany) followed by washing.

After being successively deparaffinized in xylene and hydrated in a series of ethanol, the sections of liver tissue from infected rats were blocked in 3% (v/v) H_2_O_2_ in PBS for 15 min to exhaust endogenous peroxidase. The sections were blocked with normal goat serum for 2 h at RT followed by antigen retrieval in 10 mM citrate buffer (pH 9.6) at 95°C for 30 min using a water bath. The sections were incubated with mouse anti-r*Cs*HK serum (1:100 dilution) or serum from a pre-immune mouse. After washing, the sections were probed with HRP-conjugated goat anti-mouse IgG (1:400 dilution, Protein tech., USA) for 1 h at RT. The immunoreactive signal was developed by DAB reagent. At last, the sections were counterstained with Mayer’s hematoxylin, dehydrated, cleared in xylene and imaged under a light microscope (Carl Zeiss, Germany).

### Enzyme-linked immunosorbent assay (ELISA) of antibody titers and isotype of IgG induced by r*Cs*HK

Microplates were coated with 2 μg/well purified r*Cs*HK in coating buffer (0.1 M carbonate-bicarbonate, pH 9.6) and incubated at 4°C overnight. Subsequently, the plates were blocked with 5% skimmed milk in PBST for 2 h at 37°C. After washing, the wells were incubated with different dilutions of the immune serum (6 weeks after the first immunization) raised by r*Cs*HK. Serum from rats immunized with PBS was measured as a negative control. HRP-conjugated goat anti-rat IgG (1:20,000 dilution in 0.1% BSA-PBST, Protein tech., USA) was used as the secondary antibody. After incubation for 1 h and washing three times with PBST, the reactions were developed by adding 100 μl of substrate solution (TMB, BD biosciences, San Diego, USA) followed by 10 min in darkness. The absorbance was measured at 450 nm after adding 2 M H_2_SO_4_ to stop the reaction. The levels of total IgG and IgG isotype in serum collected at different time points (0, 2, 4, 6, 8, 10, 12 weeks after the first immunization) were determined by the aforementioned process. The dilutions of the serum were 1:400. HRP-conjugated goat anti-rat IgG (1:20,000 dilution)/IgG1/IgG2a (1:10,000 dilution, Bethyl, Texas, USA) were employed as secondary antibodies.

### Culture of *C*. *sinensis* adults with rat anti-r*Cs*HK serum

Adult worms newly recovered from infected rats were washed three to four times with sterilized PBS with 1% antibiotics (penicillin 100 μg/ml and streptomycin 100 U/ml). They were then transferred to 12-well plates with 20 adults per well and incubated in 2 ml of low glucose DMEM with 1% antibiotics. Serum from r*Cs*HK immunized rats or pre-immune rats was added to the medium at dilutions of 1:160–1:40. Low glucose DMEM was used as a blank control. The worms were monitored under a microscope (Leica, Germany) for 5 min, and intact alive worms were counted at 1, 2, 3, 4, 5, 6, 7, 8, 9, 10, 15, 18, 20, 22, 24, 26, and 28 days after the incubation. Worms with no muscle contraction or no pumping after 5 consecutive shots were considered to be dead [24].

Parasites incubated in medium with diluted rat anti-r*Cs*HK serum for 1, 3, 5, and 6 days were collected. The worms were suspended and then homogenized in RIPA lysis buffer (containing 1 mM proteinase inhibitor PMSF, Bioteke, China). The supernatant was collected after centrifugation for 15 min at 10,000 × g at 4°C and the concentration of total protein was determined using a BCA protein assay kit (Novagen, USA). The enzymatic activity of native *Cs*HK in the samples was assayed as described above. The enzymatic activity of secreted phospholipase A2 from *C*. *sinensis* (*Cs*PLA_2_) was assayed as a control with the sPLA_2_ assay kit (Cayman Chemical, USA) according to the manufacturer’s instructions.

### Immune protective efficacy of r*Cs*HK

Thirty-two 6-week-old SD rats were randomly divided into four equal groups: infection group, adjuvant group, PBS group, and r*Cs*HK group. r*Cs*HK (200 μg) or an equivalent volume of PBS was emulsified with complete Freund’s adjuvant and subcutaneously injected into SD rats in the r*Cs*HK group and PBS group. r*Cs*HK (100 μg) emulsified with incomplete Freund’s adjuvant was given for the next two boosters at 2-week intervals. An equivalent volume of adjuvant was injected subcutaneously into SD rats in the adjuvant group.

After the measurement of antibody titers at week 6 post immunization, the rats (n = 8 in each group) were anesthetized with ether and intragastrically challenged with 80 live metacercariae of *C*. *sinensis*. The eggs per gram feces (EPG) was counted with a previous method [25] at 6 weeks after the infection. All rats were sacrificed at week 8 post infection to recover adult worms from their livers for worm burden evaluation. All rats were kept under the same conditions until sacrifice. EPG and worm burden were counted blindly. Reduction rates in parasite burden were calculated as follows. Worm reduction rate (%) = [(average worm burden of control group—average worm burden of experimental group) / average worm burden of control group] × 100%. Egg reduction rate (%) = [(average EPG of control group—average EPG of experimental group) / average EPG of control group] × 100%.

### Statistical analysis

All of the experiments were repeated at least three times in triplicate. SPSS version 13.0 software was used for statistical analysis. Student’s *t* test was used to analyze IgG isotypes and immune protective efficacy among the groups. The survival rates of cultured worms were determined using the Kaplan-Meier method, and differences between the groups were identified through log-rank analysis. The results are presented as mean ± SD, and *p* < 0.05 was classified as statistically significant.

## Results

### Spatial structure differences between *Cs*HK and HKs from definitive hosts of *C*. *sinensis*, humans and rats

**Fig 1:**
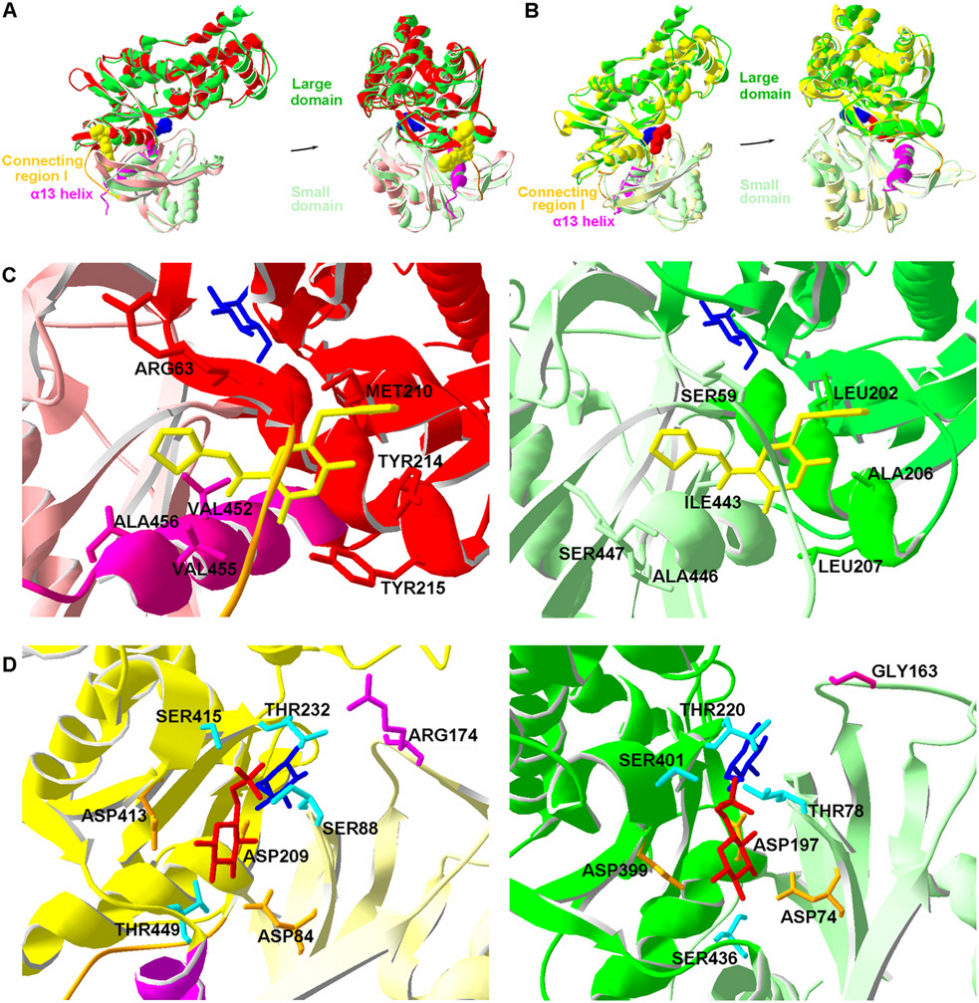
Comparison of putative spatial structure of *Cs*HK with hHK-IV or rHK-In. (A) Ribbon drawing of superposed structure models of *Cs*HK (green and light green) and closed-form hHK-IV (red and light red, PDB: 1V4S_A), which structures are complexed with glucose (blue ball) and MRK (N-thiazol-2-yl-2-amino-4-fluoro-5-(1-methylimidazol-2-yl) thiobenzamide, an allosteric activator, yellow ball). The α 13 helix (magenta and light green) is included in the small domain of the closed-form. (B) Ribbon drawing of superposed structure models of *Cs*HK (green and light green) and rHK-In (yellow and light yellow, PDB: 1BG3_B), which structures are complexed with glucose (blue ball) and G6P (red ball). The α 13 helix (magenta and light green) is included in the small domain of the closed form. The structures of the α 13 helix and connecting region I (brown and light green) are different. (C) Stereo view of the allosteric sites in closed-form hHK-IV (left) and *Cs*HK (right). In the left panel, the allosteric sites are located below connecting region I (brown, ribbon model). MRK (yellow stick) forms hydrogen bonds with ARG63 and TYR215 (red stick) and hydrophobically interacts with MET210, TYR214 (red stick) of α 5 helix (red ribbon) and V452, V455 (magenta stick) of α 13 helix (magenta ribbon). The supposed corresponding structure of *Cs*HK is shown in the right panel. (D) Stereo view of G6P binding sites in rHK-In (left) and *Cs*HK (right). Interactions of G6P (red stick) with the large (yellow) and small (light yellow) domain of the rHK-In binding cleft are shown in the left panel. SER/THR residues are colored light blue (stick), and ASP residues are orange (stick). Glucose (blue stick) is bound at an adjacent position in the cleft. The ARG174 side chain unique to rHK-In is shown in magenta (stick). The supposed corresponding structure of *Cs*HK is shown in the right panel.

*Cs*HK is composed of a large domain (green) and a small domain (light green). The two domains are linked by connecting regions I-III (light green). In closed-form *Cs*HK, the α 13 helix is included in the small domain. The lengths and amino acid residues of α 13 helix (magenta and light green) and connecting region I (brown and light green) of *Cs*HK are different from those of hHK-IV and rHK-In (Fig. 1A-1B). The allosteric sites in closed-form hHK-IV are ARG63, MET210, TYR214, TYR215, VAL452, VAL455, and ALA456. In *Cs*HK, the corresponding sites are SER59, LEU202, ALA206, LEU207, ILE443, ALA446, and SER447 (Fig. 1C). As for G6P binding sites of rHK-In, SER88, ARG174, and THR449 are replaced by THR78, GLY163, and SER436 in *Cs*HK (Fig. 1D).

### Effects of phosphate donors, effectors and a small molecule inhibitor (EbSe) on the enzyme kinetics of r*Cs*HK

**Fig 2:**
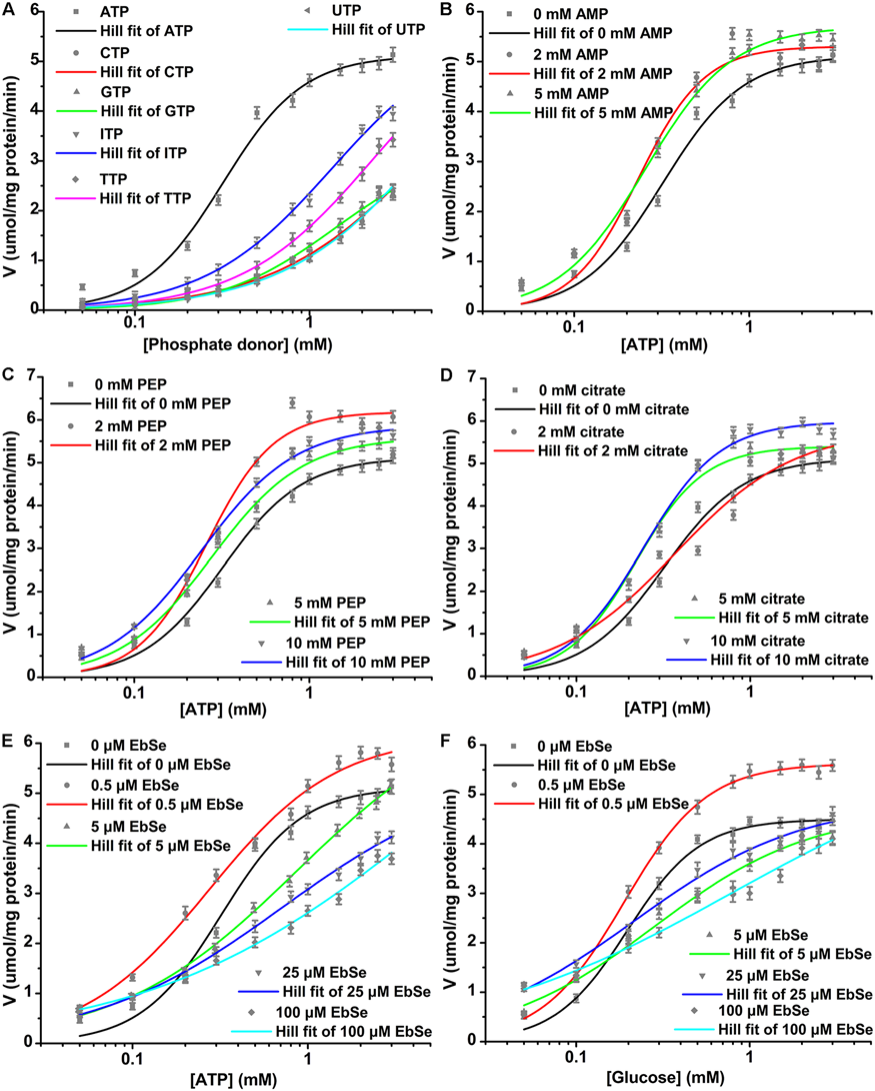
Effects of phosphate donors, effectors and EbSe on the enzyme kinetics of r*Cs*HK. The effect of 0~3 mM phosphate donors (ATP, CTP, GTP, ITP, TTP, and UTP) and fixed 3 mM glucose (A). The effect of 0~5 mM AMP (B), 0~10 mM PEP (C), 0~10 mM citrate (D), or 0~100 μM EbSe (E) and fixed 3 mM glucose with respect to ATP. The effect of 0~100 μM EbSe and fixed 3 mM ATP with respect to glucose (F).

r*Cs*HK catalyzed the phosphorylation of a series of hexoses at the following relative velocity (Table 1): D(+)-glucose (100%) congruent to D(+)-mannose (97.13%) greater than D(-)-fructose (16.60%) greater than D(+)-galactose (0.23%). With respect to phosphate donors, r*Cs*HK could use ATP, CTP, GTP, ITP, TTP, and UTP, and r*Cs*HK was less specific for ATP. Very little or no dephosphorylating activity was found for ADP, AMP, and inorganic pyrophosphate (PPi). ATP was able to be replaced by other nucleotides with moderate relative velocity. ATP, GTP, ITP and TTP homotropically and allosterically activated the enzyme (Hill coefficients, h > 1), whereas CTP homotropically and allosterically inhibited the enzyme (h < 1). UTP has no allosteric effect on the enzyme (h = 1) (Fig. 2A, S2 Table). r*Cs*HK was inhibited by high concentrations of ATP. At physiological concentration (5 mM) [14, 26], ATP showed 8% inhibition of r*Cs*HK, whereas other nucleotides showed no inhibition of r*Cs*HK. ATP, CTP, and TTP showed 13.8, 8.6, and 14.3% inhibition of r*Cs*HK at 10 mM concentration, respectively, whereas other nucleotides showed no inhibition of r*Cs*HK.

**Table 1:** Substrate specificity of r*Cs*HK.

Substrate	Relative velocity (%)
Hexose^a^	
D(+)-glucose	100.00
D(+)-mannose	97.13
D(-)-fructose	16.60
D(+)-galactose	0.23
Phosphate donor	
ATP	100.00
CTP	17.73
GTP	14.22
ITP	35.28
TTP	21.40
UTP	13.10
ADP	1.65
AMP	0.21
PPi	0.04

^a^ from reference [17].

AMP exhibited a mixed allosteric K+V+ effect [27] on r*Cs*HK by decreasing its *K*_0.5_ and increasing *V*_max_ with respect to ATP (Fig. 2B, S2 Table). PEP displayed allosteric activation of r*Cs*HK with respect to ATP with mixed K+V+ allosteric effects in a dose-independent manner (Fig. 2C, S2 Table).

Citrate exhibited an unusual mixed allosteric effect on r*Cs*HK with respect to ATP. At 5 mM and 10 mM citrate behaved as a mixed K+V+ activator, whereas at 2 mM citrate behaved as a V activator and a K inhibitor (antiergistic or crossed mixed K−V+ effect) [28] (Fig. 2D, S2 Table). Under these conditions, V activation contributed less to the effective reaction rate compared to K inhibition. The resulting effect was a net inhibition by 2 mM citrate with a reduction of h from 1.935 ± 0.271 to 1.267 ± 0.242.

At 0.5 μM EbSe behaved as a mixed K+V+ allosteric activator of r*Cs*HK with respect to ATP and glucose, whereas at 5 μM, 25 μM or 100 μM EbSe displayed net allosteric inhibition of r*Cs*HK with mixed K−V+ effects with respect to ATP and glucose in a dose-independent manner (Fig. 2E-2F, S2 Table). r*Cs*HK was not inhibited by 2 mM of D-fructose 6-phosphate or D-fructose 1,6-diphosphate.

### Western blotting analysis

**Fig 3:**
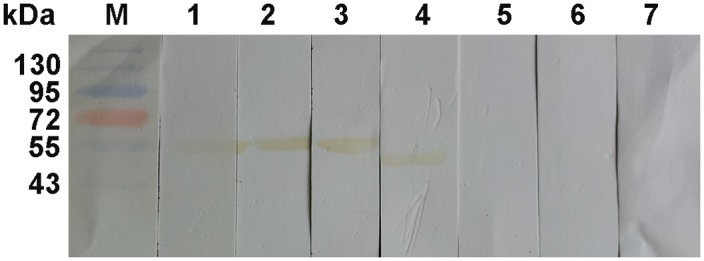
Western blotting analysis of r*Cs*HK. Pre-stained protein markers (lane M), r*Cs*HK reacted with serum from *C*. *sinensis* infected humans (lane 1), serum from *C*. *sinensis* infected rats (lane 2), or rat anti-*Cs*ESPs serum (lane 3). *Cs*ESPs were probed with anti-r*Cs*HK rat serum (lane 4). r*Cs*HK did not react with serum from healthy people (lane 5) nor with serum from pre-immune rats (lane 6), and *Cs*ESPs probed with serum from pre-immune rats (lane 7).

Purified r*Cs*HK was probed with serum from *C*. *sinensis* infected humans/rats and rat anti-*Cs*ESPs serum yielding a cross-reactive band of approximately 54.8 kDa (including molecular mass of a His-tag) [17], but it was not recognized by serum from healthy people or from a pre-immune rat. In addition, *Cs*ESPs blotted with rat anti-r*Cs*HK serum, but not with serum from a pre-immune rat, yielded a band at approximately 50.0 kDa (Fig. 3).

### Immunolocalization of *Cs*HK in *C*. *sinensi*s and in liver tissue from infected rats

**Fig 4:**
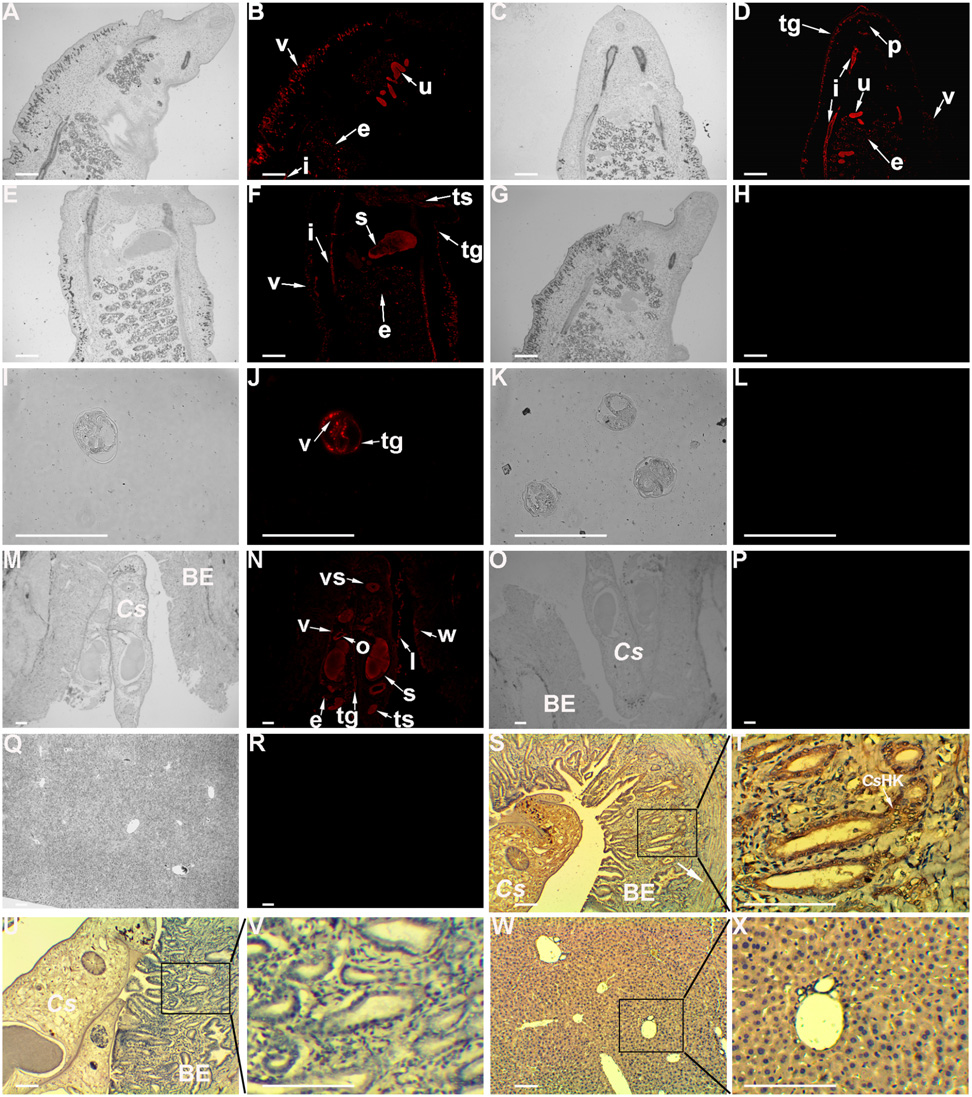
Immunolocalization of *Cs*HK in *C*. *sinensis* and in liver from infected rats. Mouse anti-r*Cs*HK serum and anti-mouse IgG were applied as primary antibody and secondary antibody, respectively. Serum from pre-immune mice was employed as primary antibody for a negative control. Panels H, L, P, R, U, V, W, and X are negative controls. Panels B, D, F, H, J, L, N, P, and R are under fluorescence microscope and the same parts (panels A, C, E, G, I, K, M, O, and Q) are under white light. Panels B, D, and F, localization of *Cs*HK in adult worms; panel J, localization of *Cs*HK in metacercariae. Panels S and T, localization of *Cs*HK in intrahepatic bile ducts of a *C*. *sinensis* infected rat. In panels S, T, U, V, W, and X, peroxidase staining shows as a yellow/rust colored deposit and Mayer’s hematoxylin counterstains the nuclei in light purple. White arrows highlight the regions of intrahepatic bile duct tissue and the tissue that stained positive for *Cs*HK. Original magnification: × 50 for panels M, N, O, P, Q and R; × 100 for panels A, B, C, D, E, F, G, H, S, U, and W; × 400 for panels I, J, K, L, T, V, and X. Bar = 800 μm. v, vitellarium; e, egg; vs, ventral sucker; tg, tegument; i, intestine; u, uterus; ts, testicle; o, ovary; p, pharynx; s, spermatheca; l, lumen; w, within the cells; *Cs*, *C*. *sinensis*; BE, biliary epithelium.

In adult worms (Fig. 4), strong fluorescence of *Cs*HK was detected in the vitellarium, tegument, intestine, spermatheca, testicle, pharynx, uterus and egg in uterus, but not in the negative control. In metacercariae, strong fluorescence was distributed in the tegument and vitellarium. In slides of liver from infected rats incubated with mouse anti-r*Cs*HK serum, strong fluorescence was detected in the vitellarium, tegument, intestine, spermatheca, testicle, ovary, ventral sucker, uterus and egg in uterus of the worms inside the bile duct. In addition, specific fluorescence was also observed in the intrahepatic biliary epithelium and lumen of the biliary tract near the parasites. No specific fluorescence was detected in the negative control incubated with serum from a pre-immune mouse.

In slides of infected liver developed for color by DAB reagent, specific brown staining was detected in the intrahepatic bile ducts with adenomatoid hyperplasia, but it was not observed in the negative control incubated with serum from a pre-immune mouse or in liver slides from normal rats incubated with mouse anti-r*Cs*HK serum.

### Rat anti-r*Cs*HK serum affects *C*. *sinensis* adult survival in vitro

**Fig 5:**
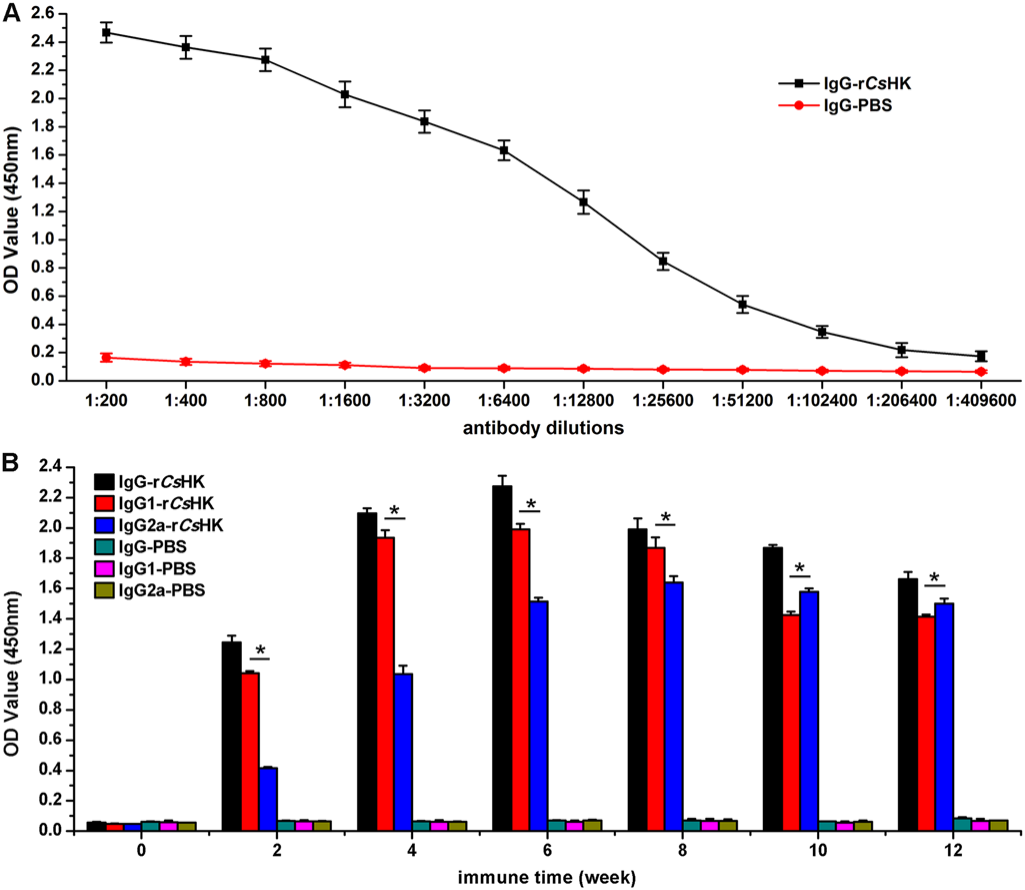
ELISA of antibody titers and isotype of IgG induced by r*Cs*HK. (A) Antibody titers of IgG induced by r*Cs*HK. (B) IgG isotype induced by r*Cs*HK. * *p* < 0.01.

The titer of anti-r*Cs*HK IgG was up to 1:409,600 at 6 weeks after the immunization, showing the high immunogenicity of r*Cs*HK (Fig. 5A). In serum from r*Cs*HK immunized rats, IgG1 and IgG2a levels increased at 2 weeks and reached their peak at 6 and 8 weeks, respectively. From 2 to 8 weeks, the IgG1 level was statistically higher than IgG2a, but it was lower at 10 and 12 weeks (Fig. 5B).

**Fig 6:**
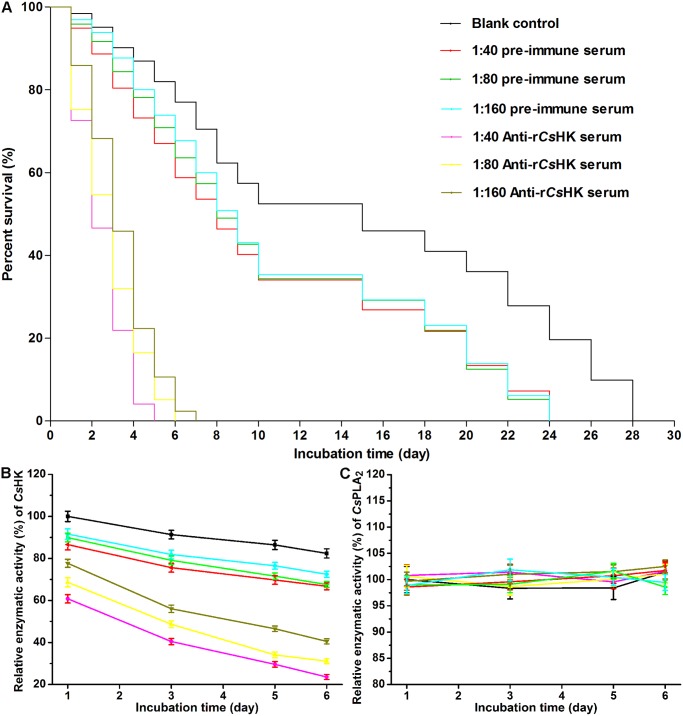
Rat anti-r*Cs*HK serum affects *C*. *sinensis* adult survival in vitro. (A) The median survival of *C*. *sinensis* adults in the blank control group, 1:40 pre-immune serum group, 1:80 pre-immune serum group, 1:160 pre-immune serum group, 1:40 anti-r*Cs*HK serum group, 1:80 anti-r*Cs*HK serum group, and 1:160 anti-r*Cs*HK serum group was 15, 8, 8, 9, 2, 3, and 3 days, respectively. There was no significant difference in survival rates among pre-immune serum groups at any dilution (*p* > 0.05). Significant differences were observed in the survival rates among the other groups (*p* < 0.05). (B) The enzymatic activity of *Cs*HK in homogenate of parasites collected from each group at 1, 3, 5, and 6 days of incubation. The enzymatic activity of *Cs*HK in adult worms incubated in medium with different dilutions of anti-r*Cs*HK serum declined significantly in a dose- and time-dependent manner. (C) As a control, there was no obvious change of the enzymatic activity of *Cs*PLA_2_ in the worms.

The median survival time of *C*. *sinensis* adults in the blank control group, 1:40 pre-immune serum group, 1:80 pre-immune serum group, 1:160 pre-immune serum group, 1:40 anti-r*Cs*HK serum group, 1:80 anti-r*Cs*HK serum group, and 1:160 anti-r*Cs*HK serum group was 15, 8, 8, 9, 2, 3, and 3 days, respectively (Fig. 6A). There was no significant difference in survival rate among the pre-immune serum groups at any dilution (*p* > 0.05). Significant differences were observed in the survival rates among all other groups (*p* < 0.05).

The enzymatic activity of *Cs*HK in adult worms incubated in medium with different dilutions of anti-r*Cs*HK serum declined significantly in a dose- and time-dependent manner (Fig. 6B). As a control, there was no obvious change in the enzymatic activity of *Cs*PLA_2_ in the worms (Fig. 6C).

### Immune protective efficacy of r*Cs*HK

The number of worms recovered in the PBS group, infection group, adjuvant group, and r*Cs*HK group was 25.1 ± 4.8, 26.1 ± 5.1, 24.8 ± 5.3, and 12.5 ± 2.4, respectively. The EPG values in the four groups were 3983.3 ± 386.7, 3895.8 ± 424.1, 4075.0 ± 473.0, and 1991.7 ± 245.4, respectively (Table 2). The worm burden and EPG were significantly lower in the r*Cs*HK group compared to the control groups (*p* < 0.01). The worm reduction rate and egg reduction rate were 50.20% and 50.00%, respectively. There was no significant difference in worm burden or EPG among the infection, adjuvant, and PBS groups.

**Table 2:** Worm burden and EPG of rats in different groups.

Group	Worm burden	Worm reduction rate (%)	EPG	Egg reduction rate (%)
PBS (n = 8)	25.1 ± 4.8		3983.3 ± 386.7	
infection (n = 8)	26.1 ± 5.1^a^		3895.8 ± 424.1^a^	
adjuvant (n = 8)	24.8 ± 5.3^a^		4075.0 ± 473.0^a^	
r*Cs*HK (n = 8)	12.5 ± 2.4^b^	50.20	1991.7 ± 245.4^b^	50.00

Results of analysis represent the mean ± SD, and the recovered worm numbers and EPG in groups were compared by Student’s *t*-test.

^a^ *p* > 0.05 and

^b^ *p* < 0.01 (compared with PBS group).

## Discussion

In the current study, we identified differences in spatial structure between *Cs*HK and HKs from the definitive hosts of *C*. *sinensis*, humans and rats. We also characterized the substrate specificity and allosteric regulation of r*Cs*HK in detail. The distribution of *Cs*HK in worms and in liver tissue and serum from *C*. *sinensis* infected rats was confirmed. Furthermore, a high-level specific antibody was induced in rats by immunization with r*Cs*HK. The enzymatic activity of *Cs*HK was suppressed by the antibody in vitro. Additionally, the survival of *C*. *sinensis* was inhibited by the antibody in vivo and in vitro.

The length and amino acid composition of the α 13 helix and of connecting region I were found to differ among *Cs*HK, hHK-IV and rHK-In. ATP-binding sites, allosteric sites, G6P binding sites and B-cell epitopes are included in these regions [17, 20, 29]. Taken together, these data suggest that the subtle structural differences between *Cs*HK and HKs from definitive hosts of *C*. *sinensis*, humans and rats, may result in remarkable changes in their enzymatic behavior.

The 100-kDa HK-I, HK-II, and HK-III of mammalian hosts have high affinity for glucose (*K*_m_ = 7–200 μM) and are strongly inhibited by G6P. The 50-kDa HK-IV, also called glucokinase, has low affinity for glucose (*K*_m_ = 5–12 mM) and is not regulated by G6P [30–32]. HK-IV, which phosphorylates glucose in liver and pancreatic islets, plays a critical role as a glucose-sensing device due to its specific regulatory properties, mainly low affinity for glucose, a sigmoidal saturation curve for this substrate, and a lack of inhibition by G6P [33–36]. Our present and previous studies [17] confirmed that r*Cs*HK is a 50-kDa G6P-sensitive allosterically modulated HK, sharing some characteristics with HKs from mammals.

Vertebrate HKs, including HK-IV, typically act on mannose, fructose and 2-deoxyglucose as well as glucose, the preferred substrate. In the rat, the four isoenzymes have essentially the same relative specificity for glucose and fructose [37]. Our results demonstrated that r*Cs*HK could use glucose, fructose, and mannose as substrates, although it preferred to use glucose and mannose. Galactose was a much poorer substrate than glucose, mannose, or fructose, in accordance with observations of HK from *Toxoplasma gondii* (*Tg*HK, a 50-kDa HK) [22]. Similarly to *Tg*HK [22], the *k*_cat_ values of r*Cs*HK for glucose (4.639 ± 0.174) and ATP (4.113 ± 0.076) were almost the same. This suggests that consumption of glucose and ATP are stoichiometrically even. However, *Tg*HK is not an allosteric enzyme [22].

Eukaryotic HKs prefer ATP as the nucleotide substrate, and *Tg*HK is no exception. r*Cs*HK showed less specificity and other nucleotides were relatively good substrates. For example, r*Cs*HK had *K*_0.5_ values of 0.315 ± 0.026 mM for ATP and 1.335 ± 0.253 mM for ITP with similar *V*_max_ values. ITP yielded 35.28% velocity relative to ATP. As for *Tg*HK, ITP yields 2.6% velocity relative to ATP [22]. By contrast, rat HK-IV, despite its much broader sugar specificity, has *K*_m_ 24-fold higher and *V*_max_ 8-fold lower for ITP than for ATP [38]. With the other isoenzymes ITP also appears to be a poor substrate [32]. When ATP, the normal phosphate donor for rat HK-IV, is replaced by ITP, the positive cooperativity with respect to glucose disappears [38]. However, both ATP (h = 1.935 ± 0.271) and ITP (h = 1.191 ± 0.109) homotropically and allosterically activated r*Cs*HK.

Fructose 6-phosphate, which is an inhibitor of yeast HK [39], does not affect the enzymatic activity of r*Cs*HK or *Tg*HK [22]. AMP exhibited a mixed allosteric K+V+ effect on r*Cs*HK by decreasing its *K*_0.5_ and increasing *V*_max_ with respect to ATP. AMP at 2 mM reduced the *V*_max_ value of *Tg*HK by 15%; however, no change in the *K*_m_ value of *Tg*HK for either glucose or ATP was observed [22].

Glycolysis is essential to *C*. *sinensis*, suggesting that enzymes involved in the pathway could be targets for drug and vaccine development [10, 40]. EbSe was identified in a screen as a potent inhibitor of *Trypanosoma brucei* HK1 (*Tb*HK1) and *Plasmodium falciparum* HK (*Pf*HK) by interrogating a selected small-molecule library of HK inhibitors [41, 42]. EbSe can promiscuously modify cysteine residues, and this nonspecific interaction is known to be the mechanism of its inhibition of some enzymes such as human indoleamine 2, 3-dioxygenase [43]. However, site-directed mutagenesis of cysteines in *Tb*HK1 and *Pf*HK did not alter their sensitivity to EbSe inhibition, indicating that either cysteine residues are not involved in EbSe inhibition or multiple cysteines must be bound in order for inhibition to occur [41, 42]. *Cs*HK shares limited sequence identity with *Tb*HK1 (36%) and *Pf*HK (31%). At 0.5 μM, 2 μM and 5 μM EbSe acts as a mixed inhibitor of *Tb*HK1 with respect to ATP [41]. However, at 0.5 μM EbSe behaved as a mixed K+V+ allosteric activator of r*Cs*HK with respect to ATP and glucose. At 5 μM, 25 μM or 100 μM EbSe displayed net allosteric inhibition of r*Cs*HK with mixed K−V+ effects with respect to ATP and glucose in a dose-independent manner. The results suggest that EbSe interacts with the two enzymes differently. EbSe has no effect on mammalian cells [41], suggesting that it may hold promise for the development of new anti-clonorchiasis compounds. Comparison of the putative spatial structure between *Cs*HK and its human and rat counterparts supports possible explanations for the significant differences in the enzymes' allosteric behavior observed in the presence of the effectors and the small molecular inhibitor, which could be exploited in drug design.

r*Cs*HK was recognized by rat anti-r*Cs*HK serum in western blotting, showing the immunoreactivity of r*Cs*HK. r*Cs*HK recognition by serum from *C*. *sinensis* infected humans/rats suggests that *Cs*HK might be a component of circulating antigens from *C*. *sinensis* [44, 45]. In addition, *Cs*ESPs were blotted with rat anti-r*Cs*HK serum, yielding a band at approximately 50 kDa. Moreover, r*Cs*HK could be recognized by rat anti-r*Cs*ESPs serum. In liver tissue from *C*. *sinensis* infected rats, immunofluorescence and immunohistochemistry showed that *Cs*HK was distributed in the intrahepatic biliary epithelium and lumen of the biliary tract near the parasites. These results indicated that *Cs*HK was also an ingredient of *Cs*ESPs.

In adult slides, *Cs*HK was extensively distributed. The locations included tegument, intestine and pharynx, where ESPs usually discharge from. The wide distribution hints that as a key enzyme involved in glycolysis, *Cs*HK is important for the worm.

*Cs*HK was observed to be expressed in the tegument. The trematode tegument is a dynamic organ involved in host-parasite interactions in addition to participating in nutrition, immune evasion and modulation, excretion, osmoregulation and signal transduction [46]. The presence of *Cs*HK in *Cs*ESPs was probably due to renewal and shedding of the tegument [47]. In trematodes, the intestine is not only a major source of secretory proteins but also a place for nutritive digestion and absorption [48]. Coupled with its localization in the tegument as a feeding structure, *Cs*HK might participate in the absorption and digestion of glucose from the host for energy supply. Moreover, the distribution of *Cs*HK in muscular tissues such as the ventral sucker and pharynx might be associated with the energy requirement for muscle contraction and adhesion behavior. Its distribution in reproductive organs such as the vitellarium, testis, spermatheca, ovary, and uterus suggests that continuous catalytic activity of *Cs*HK for glucose metabolism might take place in these organs to meet the energy demands for growth and reproduction of the parasite. The trematode vitellarium plays a key role in egg production by supplying eggshell material, relevant enzymatic activity and nutrients to the fertilized ovum [49]. The localization of *Cs*HK in eggs is consistent with the highest mRNA and protein levels of *Cs*HK occurring in the egg life stage [17]. It has been speculated that *Cs*HK plays a crucial role in maintaining glucose metabolism for the development of eggs and formation of the eggshell.

The distribution of *Cs*HK in liver tissue from *C*. *sinensis* infected rats demonstrated the abundant excretory expression profile of *Cs*HK in intrahepatic bile ducts of the host. This suggests that *Cs*HK might mediate direct interactions with host cells as a component of *Cs*ESPs, and it may derive from the excoriation of parasites and excretion through the intestine or glands [4] when *C*. *sinensis* inhabits the host. The localization of *Cs*HK on bile duct epithelial cells close to the resident worms and the surface of hyperplastic adenoma suggests that *Cs*HK might be internalized, taken up and/or translocated from the parasite by host cells.

The rapid increase of specific antibody and titers up to 1:409,600 at 6 weeks after immunization with r*Cs*HK by ELISA shows the strong immunogenicity of r*Cs*HK. Bioinformatics tools indicate an abundance of putative B-cell and T-cell epitopes in *Cs*HK [17]. The high levels of specific antibody elicited by r*Cs*HK might result from its multiple B-cell epitopes. In serum from r*Cs*HK immunized rats, IgG1 and IgG2a levels increased. It is well known that IgG2a and IgG1 are, respectively, induced by T helper cells (Th) 1 and Th2. Our results suggest that r*Cs*HK induced a combined Th1/Th2 immune response. During long-term *C*. *sinensis* infections, there is a Th1 to Th2 shift, resulting in chronic liver fluke disease and long-term survival of the worm [50]. In r*Cs*HK immunized rats, the levels of IgG1 were statistically higher than those of IgG2a from 2 to 8 weeks, but lower at 10 and 12 weeks. The rats were challenged 6 weeks after the first immunization. The worm burden and EPG in the r*Cs*HK immunized group significantly decreased compared to the control groups at 12 weeks after the first immunization. The role of Th1 cells is to orchestrate protective proinflammatory immune responses [51]. It has been documented that protected animals elicit high levels of both IgG1 and IgG2 antibodies, whereas the magnitude of these are 10-and 100-fold lower in non-protected animals. Protection is tightly correlated with the level and avidity of the IgG2 antibodies induced [52–54]. Moreover, for successful vaccination against most bacterial and viral diseases, an efficient Th1 response is required [55]. The decrease of worm burden and EPG in the r*Cs*HK immunized group might be related to the up-regulated immune responses, especially Th1, evoked by r*Cs*HK at 10 weeks post immunization.

The survival rates of *C*. *sinensis* adults incubated in medium with different concentrations of rat anti-r*Cs*HK serum statistically decreased compared to those of worms incubated in medium with pre-immune serum. The enzymatic activity of *Cs*HK in adult worms incubated in medium with different dilutions of anti-r*Cs*HK serum declined significantly in a dose- and time-dependent manner. The inhibition of *Cs*HK enzymatic activity by anti-r*Cs*HK serum might contribute to the decrease of worm burden and EPG in the r*Cs*HK immunized group.

Collectively, we confirmed that differences exist in spatial structure and affinity for hexoses and phosphate donors between *Cs*HK and HKs from humans or rats, the definitive hosts of *C*. *sinensis*. We found that effectors (AMP, PEP, and citrate) and a small molecular inhibitor regulate the enzymatic activity of r*Cs*HK with various allosteric systems. *Cs*HK was found to be extensively distributed in adult worms. It was confirmed to be a component of ESPs. r*Cs*HK showed relatively good immunogenicity and immunoreactivity. Subcutaneous immunization with r*Cs*HK decreased worm burden and EPG in challenged rats, which might be related to the up-regulated immune responses, especially Th1, evoked by r*Cs*HK and to the inhibition of *Cs*HK enzymatic activity by anti-r*Cs*HK serum. Our study showed that *Cs*HK has vaccine potential and is a promising drug target for Clonorchiasis, making it worthy of further investigation.

## Supporting Information

S1 Table Accession numbers/ID numbers for genes and proteins mentioned in the text. (XLS)

S2 Table Summarized kinetic parameters of r*Cs*HK fitting the Hill equation. (XLS)
